# Mindscapes of Neurorehabilitation: Insights from the European Congress on Neurorehabilitation 2023 – Part I

**DOI:** 10.25122/jml-2023-1029

**Published:** 2023-10

**Authors:** Cristian Andriescu, Alexandra Gherman, Stefana-Andrada Dobran, Dafin Muresanu

**Affiliations:** 1 RoNeuro Institute for Neurological Research and Diagnostic, Cluj-Napoca, Romania; 2 Sociology Department, Babes-Bolyai University, Cluj-Napoca, Romania; 3 Department of Neuroscience, Iuliu Hatieganu University of Medicine and Pharmacy, Cluj-Napoca, Romania

Rehabilitation is a core part of neurological sciences, serving as a focal point to ensure optimal clinical patient outcomes and improve the quality of life through holistic approaches. The intricate nature of neurorehabilitation comprises a multifaceted process, requiring collaborative efforts from diverse disciplines with an integrated top-down and bottom-up perspective. The synergy of technology and traditional techniques ensures that the patient receives the best possible care.

Throughout the years, the European Federation for Neurorehabilitation (EFNR) has developed an impressive network of European neurorehabilitation societies, summing up 24 member societies that represent the organization's backbone. Moreover, the EFNR is engaged in collaboration with very well-established European and international organizations such as the World Federation for Neurorehabilitation (WFNR), the World Health Organization (WHO), the European Academy of Neurology (EAN), The European Stroke Organization (ESO), the Academy for Multidisciplinary Neurotraumatology (AMN), and the World Federation of Neurology (WFN). These partnerships are built upon four pillars: education, research, medical practice, and advocacy, all converging toward the common goal of addressing the most pressing health issues of today, in neurological disorders and across the health spectrum, to ensure, within the frame of high quality of life standards, a brighter future for patients throughout the world.

**Figure 1:**
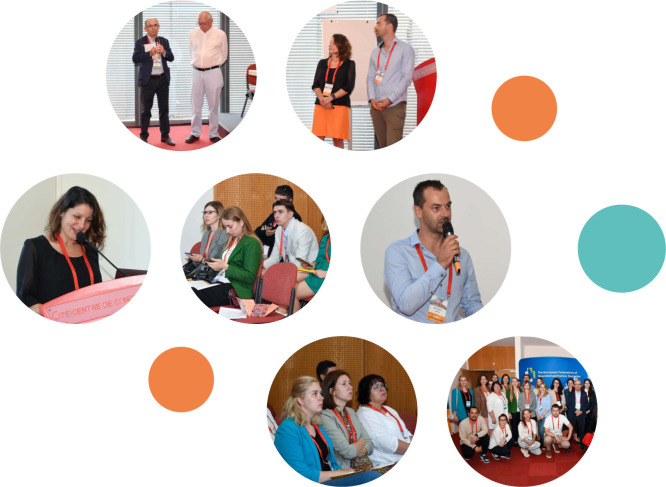
Dysphagia Course in Lyon, France (upper left & right: Prof. V. Hömberg – WFNR President & Prof. D. Muresanu – EFNR President; Dr M. Trapl-Grundschober & B. Degen - Dysphagia Course Trainers; center & bottom: images from 2^nd^ edition of Dysphagia Course Series)

The most recent example of fruitful collaboration is the Dysphagia Course Series, organized with ESO and the Stroke Alliance for Europe, which came forward to tackle one of the most common post-stroke affection and highlighted a core and the largest stroke regional project, the Stroke Action Plan for Europe (SAP-E). Following the positive feedback received after the first dysphagia course, organized in late June in Tashkent, Uzbekistan, the three organizations extended the program and developed the second edition of the course on 29 August 2023 in Lyon, France. On this occasion, 22 delegates from the member societies of EFNR had the chance to interact with world-renowned experts in the complex field of dysphagia (Figure 1), thus tailoring the course into a great success and an important landmark for the collaboration between EFNR and the international organizations involved.

**Figure 2:**
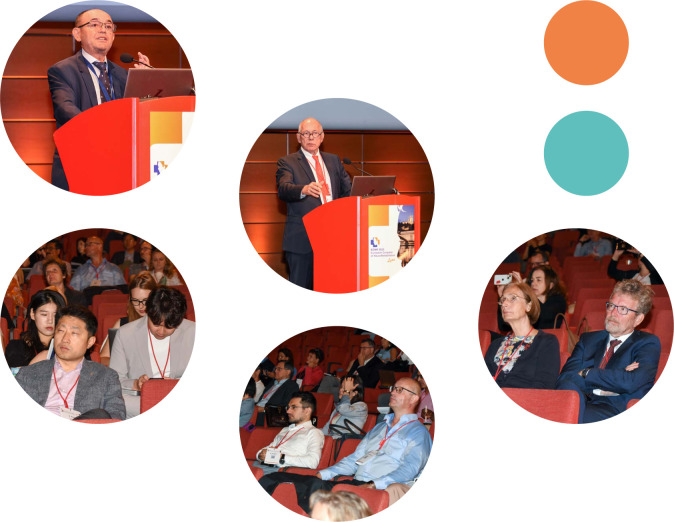
Photos from the European Congress on Neurorehabilitation 2023

The course was followed by one of the largest congresses of this Autumn - the 7^th^ edition of the European Congress on Neurorehabilitation (ECNR). The four-day hybrid event took place between 30 August and 3 September in Lyon, France, and it was organized by the European Federation of Neurorehabilitation Societies (EFNR) in collaboration with The French Society of Physical and Rehabilitation Medicine (SOFMER). The charming French city became home to nearly 800 participants and speakers from 57 countries (Figure 2), offering an outstanding international dimension to the event, which put forward a mind-provoking glimpse into the realities and prospects of neurorehabilitation at the European level but also in the broader, global context.

ECNRs are biannual events organized by the European Federation of Neurorehabilitation Societies, and they focus on bringing together scientists, clinicians, therapists, and patient organizations, as well as political representatives, from a vast array of disciplines in the field of neurorehabilitation, to work together on developing environments oriented toward patient care.

**Figure 3:**
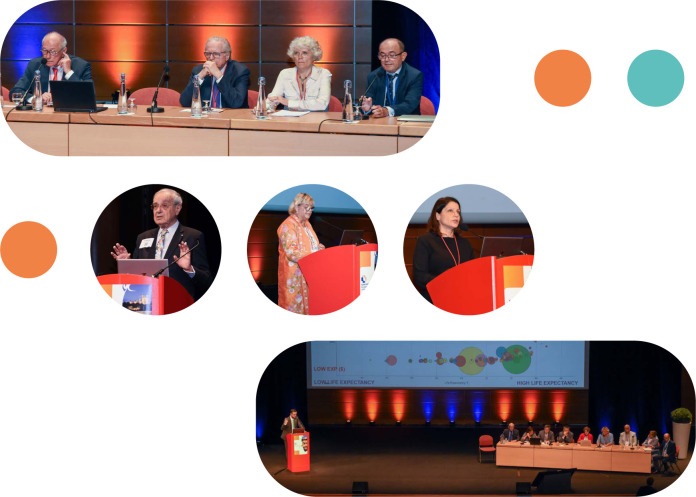
Speakers from the European Congress on Neurorehabilitation 2023 (upper left, from left to right: Prof. V. Hömberg - WFNR President; Prof. H. Binder - Honorary and Founding Member of EFNR; Prof. Isabelle Bonan - President of SOFMER; Prof. D. Muresanu - EFNR President; center, from left to right: Distinguished Professor V. Hachinski, Prof. M. Leonardi - WHO Representative, Dr. M. Trapl-Grundschober - Dysphagia Trainer; bottom right; image from the roundtable on Public Health Policies)

The 2023 scientific program included 13 workshops, 7 plenary lectures, 28 symposiums (out of which 7 were in cooperation with partner societies), 121 invited speakers (Figure 3), 109 ePoster sessions, and 2 roundtables.

The Opening Ceremony, hosted by Prof. Dafin Muresanu, President of EFNR, welcomed numerous distinguished personalities to the podium. Prof. Volker Hömberg (President of WFNR), Prof. Wolfgang Grisold (President of World Federation of Neurology), Prof. Matilde Leonardi (Institutional delegate at WHO), Prof. Francesca Pezzella (Chairperson of SAP-E), Prof. Johannes Vester (President of the AMN), Heinrich Binder (Founding President of EFNR), and Isabelle Bonan (President of SOFMER), all offered a warm welcome address to the audience.

Prof. Matilde Leonardi, the liaison with the WHO, shared key points on the activity of the World Health Organization regarding neurorehabilitation and ensuring global health, placing a highlight on the important need to address the rights of people with disability, identify barriers to care, and take urgent action to deliver accessible rehabilitation. Moreover, improving the quality of patients’ lives should be considered in the context of the bio-psycho-social model of health and disability, the best indicator of treatment needs and outcomes. She discussed about pursuits of the WHO, such as the International Classification of Diseases (ICD) 11. As the health crisis has biomedical, social, political, economic, and cultural dimensions, the burden of neurological disorders is increasing (both in mortality and morbidity), and health systems worldwide face crises and lack of rehabilitation insomuch that directed action is needed. The Intersectoral Global Action Plan for Epilepsy and other Neurological Disorders (IGAP) aims to reduce the stigma and impact of neurological disorders to reach brain health. Last but not least, the Rehabilitation 2030 WHO Plan was addressed, a strategy directed at improving the integration of rehabilitation in the health sector and strengthening intersectoral links to meet population needs effectively and efficiently and incorporate rehabilitation into universal health coverage.

Further on, Prof. Dafin Muresanu discussed the collaboration between the EFNR and WFNR, working hand in hand with the EAN, highlighting the importance of IGAP and the Rehabilitation 2030 Initiative to create awareness and mobilize decision-makers. Community-based rehabilitation programs are a key element in ensuring better patient outcomes globally, as they reduce the burden on centralized healthcare systems. However, it is important to consider the steps for implementation, identify gaps, establish partnership and capacity, as well as to develop and implement monitoring, evaluation and capitalization activities.

Also, the EFNR president proposed plans to expand the network in Central Asia, following the WFNR-EFNR meeting in Tashkent and outlined the importance of the National (Romanian) Strategy for Cardiovascular and Cerebrovascular Diseases within the frame of guideline development and patient care. Professor D. Muresanu highlighted collaborative projects such as the Neurotrauma Treatment Simulation Center in Vienna, the memorandum of understanding with ESO, the Teaching Courses on Dysphagia, the guidelines developed together with the EAN on pharmacological support on early motor rehabilitation, and the partnership with WHO on the Brain Health Initiative.

At the end of the Opening Ceremony, on behalf of WFNR, Professor Volker Hömberg discussed the activities of the World Federation for Neuro Rehabilitation, with a focus on the mentorship program for younger clinicians and the over 40 Special Interest Groups developed, which promote the growth of their area of interest, and especially their involvement in educational activities.

Further insights from the 7^th^ European Congress on Neurorehabilitation shall be presented in part II of this article, which will provide the readers with details from the plenary sessions, special joint EFNR – partner societies sessions, and concluding remarks from the ECNR 2023 in Lyon, France.

